# Association of single nucleotide polymorphisms of *MTHFR, TCN2, RNF213* with susceptibility to hypertension and blood pressure

**DOI:** 10.1042/BSR20191454

**Published:** 2019-12-17

**Authors:** Shan Liu, Mengwei Liu, Qian Li, Xiuping Liu, Yue Wang, Michael Mambiya, Kaili Zhang, Luping Yang, Qian Zhang, Mengke Shang, Fanxin Zeng, Fangfang Nie, Wanyang Liu

**Affiliations:** Department of Nutrition and Food Hygiene, School of Public Health, China Medical University, Shenyang, China

**Keywords:** gene polymorphisms, Hypertension, MTHFR, RNF213, TCN2

## Abstract

Methylenetetrahydrofolate reductase gene (*MTHFR*), transcobalamin*II* (*TCN2*) and ring finger protein 213 (*RNF213*) are related to homocysteine (Hcy) level and are of great significance for hypertension. We aimed to evaluate the associations of *MTHFR* (rs1801133, rs1801131, rs9651118), *TCN2* (rs117353193) and *RNF213* (rs9916351) with hypertension and blood pressure (BP). A total of 953 patients with hypertension and 1103 controls were enrolled. Genotyping was performed by Taqman. Logistic regression analysis indicated that A allele of *TCN2* rs117353193 under the dominant model had a significantly protective effect (*P*=0.045) after adjustment, which showed that AA+GA genotype has a lower risk than GG. Additionally, the average diastolic BP (DBP) (*P*=0.044) and mean arterial pressure (MAP) (*P*=0.035) levels were significantly different between genotypes of *RNF213* rs9916351. Further pairwise comparison showed that the average systolic BP (SBP) level of the TT genotype carriers were significantly higher than in CC (*P*=0.024), and the average DBP and MAP levels of the TT genotype carriers were higher than in CT (*P*=0.044, *P*=0.012, respectively) and CC (*P*=0.048, *P*=0.010, respectively). In the recessive model, the average SBP (*P*=0.043), DBP (*P*=0.018) and MAP (*P*=0.017) levels with the TT genotype carriers were significantly higher than in CT+CC. Multiple linear regression analysis suggested that *RNF213* rs9916351 in the recessive model had significant effects on SBP (*P*=0.025), DBP (*P*=0.017) and MAP (*P*=0.010) as a risk factor. However, no associations were observed between *MTHFR* and hypertension. *TCN2* rs117353193 might serve as a protective factor in hypertension, and *RNF213* rs9916351 might be a risk factor that is linked to increase BP level in Northeast Chinese population.

## Introduction

Hypertension is a multifactorial disease and is a major life-threatening health concern throughout the world. Approximately 2 million people in China die of diseases directly associated with hypertension each year, and its the prevalence rate is still on the rise. Especially in the middle-aged and old population, high blood pressure (BP) has become the main cause of coronary heart disease, stroke and many other cardiovascular diseases (CVDs). The results of an 8-year follow-up with 170000 people over 40 years old in China showed that coronary heart disease was the first cause factor of death, while hypertension was the first risk factor for total mortality, and the relative risk ratio (RR) was 1.48 [1]. The pathogenesis of hypertension is known as a result of the interaction of lifestyle exposures, such as high dietary sodium, overweight and excess alcohol consumption [2]. However, previous studies have shown that up to 60% of the variation in inducing increased hypertension risk could be due to genetic factors [3].

A number of previous studies have suggested that genetic alterations in the genes controlling homocysteine (Hcy) and folate metabolism were linked to onset of CVDs [4,5]. Hyperhomocysteinemia (HHcy), an important and independent risk factor, which also contributes to endothelial damage and oxidative stress [6], has been linked to hypertension as it induces arteriolar constriction, renal dysfunction and increase in sodium reabsorption [7]. The excessive increase in Hcy level was mainly caused by gene mutation of key enzymes in the metabolic pathways.

Methylenetetrahydrofolate reductase gene (*MTHFR*), which catalyzes the conversion of 5,10-methylenetetrahydrofolate into 5-methyltetrahydrofolate, is a crucial enzyme in the metabolism of Hcy and folate which both have associated with methylation of genomic DNA [8]. Two common single nucleotide polymorphisms (SNPs), C677T (rs1801133) and A1298C (rs1801131), are particularly reported to be associated with reduced enzyme activity and thermostability, resulting in a relative deficiency in the re-methylation process and interfering with the metabolic pathway. The C/T variant at site 677 is to replace the encoded alanine with valine, and the A/C variant at site 1298, is to convert its encoded glutamic acid into alanine, which leads to elevated plasma Hcy [9–14] and damages the integrity of blood vessels [15]. Furthermore, recent evidence has found that *MTHFR* rs9651118 was associated with the serum level of Hcy and also contributes to the development of vascular diseases [16], the same is true of transcobalamin*II* (*TCN2*) rs117353193 and *RNF213* rs9916351. However, rs9651118 is an intron variant that does not cause amino acid changes.

Vitamin B_12_ is considered as a nutritional factor for regulating Hcy metabolism, the absorption and cellular delivery of which largely depend on the specific plasma transporter, *TCN2* [17]. Vitamin B_12_ and *TCN2* combine to form holotranscobalamin (holo-TC) complexus, which plays an important role in cells within target tissues [18]. The variant in its loci rs117353193 causes the encoded arginine to be converted into glutamine. Because the biological function of *TCN2* is mainly regulated by its own genetic polymorphism, it is also one of the important genetic factors affecting the metabolism of Hcy.

Ring finger protein 213 (*RNF213*) was originally identified as a susceptibility gene for moyamoya disease (MMD) [19]. MMD is often accompanied by hypertension [20,21], and the incidence of hypertension in MMD patients is significantly higher than that of the general population, suggesting that there may be a common susceptibility gene between the two. Rs9916351, though, is also an intron variant that does not cause amino acid changes. However, little data were so far found concerning the link of hypertension.

The loci of *MTHFR, TCN2* and *RNF213* have been found to be related to Hcy level in the study of susceptibility genes to MMD, which provides an idea for our study to see if there is actually a susceptibility gene loci between the two diseases. Many genome-wide association studies have been done on hypertension in different ethnic and regional populations [22,23], and the identified novel loci differ, requiring further verification. The present study was conducted to examine the association of *MTHFR* rs1801133, rs1801131, rs9651118, *TCN2* rs117353193 and *RNF213* rs9916351 gene polymorphisms with the risk of hypertension and BP in Northeast Chinese population.

## Materials and methods

### Editorial policies and ethical considerations

Before data collection, all subjects gave written informed consent in accordance with the Declaration of Helsinki. The protocol was approved by Ethics Committee of China Medical University.

### Study population

The sample size was determined using the software Power and Sample Size (PS) for calculation. The relevant parameters of the statistical efficiency calculation in the present study were as follows: (1) the lowest allele frequencies of each SNP in the Chinese population referred to the dbSNP website, where the frequency of *MTHFR* rs1801131 was set at 0.219, the frequency of rs1801133 was set at 0.333, the frequency of rs9651118 was set at 0.267, the frequency of *TCN2* rs117353193 was set at 0.033, the frequency of *RNF213* rs9916351 was set at 0.329; (2) their odds ratios (ORs) were estimated based on previous observations [16,24,25], in which the OR value of *MTHFR* rs1801131 was set to 1.56, the OR value of rs1801133 was set to 1.38, the OR value of rs9651118 was set to 0.65, the OR value of *TCN2* rs117353193 was set to 1.87, and the OR value of *RNF213* rs9916351 was set to 1.96. Among them, *MTHFR* rs9651118, *TCN2* rs117353193 and *RNF213* rs9916351 were recently found in the study of genome-wide association for MMD, there is no correlation of hypertension, so the OR value setting adopts their OR value in MMD study; (3) the ratio of sample size of control group to case group was set at 1:1; (4) test level α was set to 0.05. From the sample size calculation: 416 cases were needed for rs1801131 to be able to reject the null hypothesis reaching at least 80% power in the present study, 651 cases for rs1801133, 485 cases for rs9651118, 954 cases for rs117353193 and 145 cases for rs9916351. Meanwhile, the results of power calculation based on our sample size show that: 98.9% power for rs1801131, 92.4% power for rs1801133, 97.6% power for rs9651118, 80.0% power for rs117353193, 100% power for rs9916351.

A total of 953 patients with hypertension and 1103 controls were enrolled from Fushun and Panjin City in Liaoning Province, China. The population proportions from the two cities were not significantly different in the case and control groups (*P*>0.05). There were 894 male (43.5%) and 1162 female (56.5%) in our study. In summary, participants with hypertension who met the following criteria were recruited: (1) systolic BP (SBP) of at least 140 mmHg or diastolic BP (DBP) of at least 90 mmHg were measured three times on different days in resting state; (2) people who had been treated with antihypertensive drugs. The control group was normotensive after medical measurement (SBP < 140 mmHg and DBP < 90 mmHg). Both groups were 18 years of age and older and excluded severe liver, kidney and acute or chronic infectious diseases, hyperthyroidism or hypothyroidism, systemic arteriopathy, various tumors and other cerebrovascular diseases and metabolic diseases.

### Data collection and clinical evaluation

Clinical data including gender, age, height, weight, body mass index (BMI), waistline and smoking history were recorded in health datasheet. After sitting for 5 min, baseline BP was measured three times using a standardized mercury-gravity monometer with a 30-s interval between replicates, and the mean value of three measurements was taken. Pulse pressure (PP) was calculated as the difference of SBP and DBP. Mean arterial pressure (MAP) was calculated as the sum of one-third SBP and two-thirds DBP. Ten milliliters of peripheral blood of each fasting study individual was collected in EDTA vacutainer. Biochemical profiles, including fasting blood glucose (FBG), total cholesterol (TC) and triglyceride (TG) were done on automated biochemical analyzer (Murray, BS-820).

### DNA isolation and genotyping

After receiving informed consent, 10 ml peripheral vein blood without centrifugation was extracted from available hypertensive patients and normal control subjects placed in EDTANa_4_ anticoagulant tubes, and stored in a freezer at −80°C until analysis. Genomic DNA was extracted from blood samples with a Blood Genetic DNA Mini Kit (CWBIO, Beijing, China). The concentration of the 2056 DNA was tested by NanoDrop 2000 (Thermo Fisher Scientific, Waltham, MA, U.S.A.), of which the purity is considered to be up to the requirements of subsequent tests, then stored at −80°C for future genotyping.

Genotyping of five SNPs in all participants was conducted using Taqman™ Probe (Taqman™ SNP Genotyping Assays; Applied Biosystems, Foster City, CA, U.S.A.) and a QuantStudio™ 6 Flex Real-Time PCR System (Applied Biosystems, Foster City, U.S.A.) in a single lab. The total system contained 5 μl, 2.0 μl purified genomic DNA, 2.5 μl of TaqPath™ ProAmp™ Master Mixes (Applied Biosystems, Foster City, CA, U.S.A.), 0.1 μl of 40× SNP Genotyping Assay and 0.4 μl deoxyribonuclase-free water. The appropriate PCR thermal cycling conditions were as follows: maintained for 5 min for initial denature/enzyme activation, 40 cycles of 5 s at 95°C for denaturation, and 1 min at 60°C for annealing and extension. After each PCR amplification, an end point plate read was conducted using QuantStudio™ 6Flex Real-Time PCR System. The genotype of each sample was confirmed based on the fluorescence signals. We sampled DNA and repeated genotyping, and the results were consistent with previous experiments.

### Statistical analysis

The Epidata 3.1 software package was used for database design, data entry and data check, and SBP and DBP were respectively increased by 10 and 5 mmHg in patients with hypertension treated with drugs [26]. Statistical analysis was performed with SPSS 21.0 software. Quantitative variables were tested for normality and homoscedasticity. Those conforming to normal distributions were expressed as mean ± standard deviation, while skewed distributions were expressed as median (P_25_–P_75_). The comparison between the two groups was carried out by independent-samples *t* test or nonparametric test, and one-way analysis of variance (ANOVA) was used in the multi-group comparison. Pairwise comparison was performed by least significant difference (LSD) and student newman keuls (SNK-q) test. Qualitative variables were compared chi-square test or Fisher’s exact test and expressed as counts and proportions. Univariate logistic regression analysis was used to test the association between each SNP and hypertension under the genetic models. Binary regression analysis was used to test the association between environmental factors and hypertension. Furthermore, the multivariate logistic regression analysis was used to test the susceptibility of each SNP to hypertension after correcting the confounding factors. Multiple linear regression analysis was used to see the effects of other covariates and genetic component on BP. The SHEsis [27] software was used for Hardy–Weinberg balance test, allele and genotype correlation analysis with hypertension and haploid analysis. A value of *P*<0.05 was considered as statistically significant.

## Results

### Baseline characteristics

The clinical and demographic characteristics of 953 patients and 1103 controls are reported in Table 1. Compared with controls, the patients had significant differences in gender, age, weight, waistline, BMI, smoking frequency and higher levels of SBP, DBP, PP, MAP, FBG, TC and TG. All of these foregoing parameters were statistically higher in patients when compared with control subjects (*P*<0.05).However, no statistically significant differences were observed in height (*P*=0.864) between the two groups.

**Table 1:** Clinical and demographic characteristics of the study subjects

Characteristics	Patients (*n*=953)	Controls (*n*=1103)	*P*-value
Male:Female	439:514	455:648	**0.029^1^**
Age (years)	66 (49–71)	44 (32–66)	**<0.001^1^**
Height (cm)	162.44 ± 7.56	162.52 ± 6.93	0.864
Weight (kg)	63.15 ± 9.25	60.89 ± 8.57	**<0.001^1^**
Waistline (cm)	82.83 ± 9.01	80.03 ± 6.76	**<0.001^1^**
BMI (kg/m^2^)	23.92 ± 3.09	23.02 ± 2.84	**<0.001^1^**
SBP (mmHg)	150.68 ± 15.20	121.62 ± 11.32	**<0.001^1^**
DBP (mmHg)	91.05 ± 9.34	77.98 ± 6.80	**<0.001^1^**
PP (mmHg)	59.63 ± 14.87	43.64 ± 9.25	**<0.001^1^**
MAP (mmHg)	110.94 ± 9.28	92.51 ± 7.40	**<0.001^1^**
Smoking (%)	16.80%	7.75%	**<0.001^1^**
FBG (mmol/l)	5.60 ± 1.86	5.17 ± 1.20	**<0.001^1^**
TC (mmol/l)	4.80 ± 1.99	4.45 ± 1.20	**<0.001^1^**
TG (mmol/l)	1.78 ± 1.10	1.51 ± 1.20	**<0.001^1^**

^1^Significant difference (*P*<0.05).

### The distributions of genotypes, alleles and associations with hypertension

Genotypes and alleles frequencies of five SNPs in patients and controls are shown in Table 2. The observed genotype distributions of five SNPs among controls were in agreement with Hardy–Weinberg equilibrium (*P*=0.503 for rs1801131; *P*=0.151 for rs1801133; *P*=0.707 for rs9651118; *P*=0.555 for rs117353193; *P*=0.545 for rs9916351). However, the genotypes’ distributions and the alleles frequencies were not statistically different between the two groups (*P*>0.05).

**Table 2:** Genotypes and alleles frequency of five SNPs in patients with hypertension and controls

Genotypes and alleles	Patients (%)	Controls (%)	OR (95% CI)	*P*-value
**rs1801131**				
A	1587 (85.3%)	1852 (86.2%)	0.93 (0.78–1.11)	0.147
C	273 (14.7%)	296 (13.8%)		
AA	679 (73.0%)	801 (74.6%)		0.720
AC	229 (24.6%)	250 (23.3%)		
CC	22 (2.4%)	23 (2.1%)		
**rs1801133**				
C	839 (44.9%)	933 (43.4%)	1.06 (0.94–1.20)	0.333
T	1029 (55.1%)	1217 (56.6%)		
CC	200 (21.4%)	214 (19.9%)		0.634
CT	439 (47.0%)	505 (47.0%)		
TT	295 (31.6%)	356 (33.1%)		
**rs9651118**				
T	1383 (74.2%)	1564 (73.2%)	0.95 (0.83–1.10)	0.485
C	481 (25.8%)	572 (26.8%)		
TT	517 (55.5%)	575 (53.8%)		0.764
TC	349 (37.4%)	414 (38.8%)		
CC	66 (7.1%)	79 (7.4%)		
**rs117353193**				
G	1774 (94.9%)	2041 (94.8%)	0.98 (0.74–1.29)	0.873
A	96 (5.1%)	113 (5.2%)		
GG	840 (89.8%)	966 (89.7%)		0.900
GA	94 (10.1%)	109 (10.1%)		
AA	1 (0.1%)	2 (0.2%)		
**rs9916351**				
C	1087 (58.3%)	1195 (55.6%)	1.12 (0.98–1.26)	0.088
T	779 (41.7%)	955 (44.4%)		
CC	319 (34.2%)	337 (31.3%)		0.236
CT	449 (48.1%)	521 (48.5%)		
TT	165 (17.7%)	217 (20.2%)		

*P* is calculated for carriers of the polymorphism. Abbreviation: CI, confidence interval.

### Logistic regression analysis of environmental and genetic factors

The results of binary regression analysis of environmental factors, including gender, age, weight, waistline, BMI, smoking frequency, FBG, TC and TG, are shown in Table 3. We found that waistline (*P*=0.006) and BMI (*P*=0.016) were risk factors associated with hypertension. The results of logistic regression analysis of genetic factors are shown in Table 4. Univariate logistic regression analysis showed that the five SNPs had no significant differences under the three genetic models. After adjusting for important confounding factors, including gender, age, waistline, BMI, smoking, FBG, TC and TG, the results showed that A allele carriers of *TCN2* rs117353193 under the dominant genetic model (AA+GA vs GG) had a significantly protective effect compared with the risk of hypertension [OR = 0.56, 95% confidence interval (CI) (0.32–0.99); *P*=0.045], and the AA+GA genotype carriers had 0.56-times higher risk than the GG genotype carriers. Additionally, a borderline significant association was observed under the additive model (GA vs GG) after adjustment [OR = 0.59, 95% CI (0.33–1.04); *P*=0.069]. However, in the adjusted analysis, the effects of waistline and BMI still exist.

**Table 3:** Binary regression analysis of environmental factors

Environmental factors	OR (95% CI)	*P*-value
Gender (%)	1.129 (0.747–1.705)	0.565
Age (years)	1.007 (0.978–1.037)	0.632
Weight (kg)	0.988 (0.953–1.025)	0.528
Waistline (cm)	1.033 (1.010–1.058)	**0.006^1^**
BMI (kg/m^2^)	1.139 (1.025–1.267)	**0.016^1^**
Smoking (%)	1.556 (0.962–2.520)	0.072
FBG (mmol/l)	1.058 (0.967–1.157)	0.217
TC (mmol/l)	0.938 (0.833–1.057)	0.294
TG (mmol/l)	0.964 (0.863–1.077)	0.521

^1^Significant difference (*P*<0.05).

**Table 4:** Logistic regression analysis of five SNPs in three genetic models

SNP	Genetic model		Univariate		Adjusted^1^	
			OR (95% CI)	*P*-value	OR (95% CI)	*P*-value
**rs1801131**	**Additive**	CC vs AA	1.13 (0.62–2.04)	0.690	1.32 (0.45–3.86)	0.608
		AC vs AA	1.08 (0.88–1.33)	0.462	0.92 (0.64–1.33)	0.667
	**Dominant**	CC+AC vs AA	1.09 (0.89–1.32)	0.425	0.95 (0.67–1.35)	0.782
	**Recessive**	CC vs AC+AA	1.11 (0.61–2.00)	0.736	1.35 (0.47–3.92)	0.579
**rs1801133**	**Additive**	TT vs CC	0.89 (0.69–1.14)	0.340	1.24 (0.81–1.92)	0.324
		CT vs CC	0.93 (0.74–1.17)	0.540	1.38 (0.92–2.06)	0.117
	**Dominant**	TT+CT vs CC	0.91 (0.74–1.17)	0.405	1.32 (0.91–1.93)	0.143
	**Recessive**	TT vs CT+CC	0.93 (0.77–1.13)	0.464	1.00 (0.71–1.40)	0.999
**rs9651118**	**Additive**	CC vs TT	0.93 (0.66–1.32)	0.679	0.77 (0.41–1.46)	0.425
		TC vs TT	0.94 (0.78–1.13)	0.496	0.81 (0.58–1.12)	0.196
	**Dominant**	CC+TC vs TT	0.94 (0.79–1.12)	0.464	0.80 (0.59–1.10)	0.166
	**Recessive**	CC vs TC+TT	0.95 (0.68–1.34)	0.786	0.85 (0.45–1.58)	0.601
**rs117353193**	**Additive**	AA vs GG	-	-	-	-
		GA vs GG	0.99 (0.74–1.33)	0.955	0.59 (0.33–1.04)	0.069
	**Dominant**	AA+GA vs GG	0.98 (0.74–1.31)	0.910	0.56 (0.32–0.99)	**0.045**^**2**^
	**Recessive**	AA vs GA+GG	-	-	-	-
**rs9916351**	**Additive**	TT vs CC	1.25 (0.97–1.61)	0.091	1.13 (0.68–1.89)	0.644
		CT vs CC	1.13 (0.89–1.44)	0.304	0.86 (0.61–1.20)	0.552
	**Dominant**	TT+CT vs CC	0.88 (0.73–1.06)	0.176	0.91 (0.66–1.25)	0.552
	**Recessive**	TT vs CT+CC	0.85 (0.68–1.06)	0.155	1.23 (0.76–1.99)	0.398

1 Adjusted for gender, age, waistline, BMI, smoking, FBG, TC and TG. ^2^Significant difference (*P*<0.05).

### Haplotype distribution of three SNPs of *MTHFR* gene

Haplotype analysis of rs1801131, rs1801133 and rs9651118 polymorphisms of *MTHFR* gene are represented in Table 5. No significant differences were observed in any of the examined haplotypes (ACC, ACT, ATT, CCT) between hypertensive patients and controls (*P*>0.05). These findings suggest that the haplotypes of the *MTHFR* gene are not associated with susceptibility of hypertension in our subjects.

**Table 5:** Haplotype distribution of three SNPs of *MTHFR* gene

Haplotype	Patients (%)	Controls (%)	OR (95% CI)	*P*-value
ACC	462.58 (25.1%)	548.54 (25.9%)	0.96 (0.83–1.11)	0.576
ACT	95.85 (5.2%)	87.92 (4.1%)	1.27 (0.94–1.71)	0.115
ATT	1000.58 (54.4%)	1178.35 (55.6%)	0.95 (0.84–1.08)	0.412
CCT	264.13 (14.4%)	282.32 (13.3%)	1.09 (0.91–1.31)	0.355

All those *P*<0.05 will be ignored in analysis.

### Comparison of BP levels of five SNPs in three genetic models

As shown in Table 6, we found that the average DBP (*P*=0.044) and MAP (*P*=0.035) levels of *RNF213* rs9916351 were significantly different under the additive model, and the average SBP level had a borderline significant difference (*P*=0.077). Further pairwise comparison showed that the average SBP level with the homozygous TT genotype carriers were significantly higher than in CC genotype carriers (*P*=0.024), the average DBP and MAP levels with the homozygous TT genotype carriers were significantly higher than in CT (*P*=0.044 for DBP, *P*=0.012 for MAP) and CC (*P*=0.048 for DBP, *P*=0.010 for MAP) genotypes carriers. In the recessive model, the average SBP, DBP and MAP levels of *RNF213* rs9916351 with the homozygous TT genotype carriers were significantly higher than in CT+CC genotype carriers (*P*=0.043 for SBP, *P*=0.018 for DBP, *P*=0.017 for MAP). However, there were no significant differences in BP levels of rs1801131, rs1801133, rs9651118 and rs117353193 under the three genetic models.

**Table 6:** Comparison of BP levels of five SNPs in three genetic models

SNP	Genetic model		*n*	SBP (Mean ± SD)	*P-*value	DBP (Mean ± SD)	*P-*value	PP (Mean ± SD)	*P-*value	MAP (Mean ± SD)	*P-*value
**rs1801131**	**Additive**	CC	24	138.58 ± 17.63	0.711	87.38 ± 11.77	0.572	51.21 ± 13.48	0.599	104.42 ± 12.50	0.689
		AC	267	136.67 ± 18.22		85.01 ± 9.18		51.66 ± 14.73		102.24 ± 10.90	
		AA	754	137.77 ± 20.48		85.12 ± 10.95		52.65 ± 15.00		102.67 ± 13.05	
	**Dominant**	CC+AC	291	136.82 ± 18.15	0.466	85.21 ± 9.42	0.902	51.62 ± 14.61	0.316	102.42 ± 11.03	0.760
		AA	754	137.77 ± 20.48		85.12 ± 10.95		52.65 ± 15.00		102.67 ± 13.05	
	**Recessive**	CC	24	138.58 ± 17.63	0.789	87.38 ± 11.77	0.295	51.21 ± 13.48	0.701	104.42 ± 12.50	0.472
		AC+AA	1021	137.48 ± 19.91		85.09 ± 10.51		52.39 ± 14.93		102.56 ± 12.52	
**rs1801133**	**Additive**	TT	307	136.59 ± 19.20	0.655	84.48 ± 10.83	0.392	52.11 ± 14.41	0.914	101.86 ± 12.49	0.469
		CT	520	137.78 ± 20.60		85.24 ± 10.46		52.54 ± 15.15		102.75 ± 12.80	
		CC	223	137.92 ± 18.92		85.70 ± 10.25		52.23 ± 14.98		103.11 ± 11.80	
	**Dominant**	TT+CT	827	137.34 ± 20.09	0.696	84.96 ± 10.60	0.353	52.38 ± 14.87	0.892	102.42 ± 12.69	0.464
		CC	223	137.92 ± 18.92		85.70 ± 10.25		52.23 ± 14.98		103.11 ± 11.80	
	**Recessive**	TT	307	136.59 ± 19.20	0.359	84.48 ± 10.83	0.209	52.11 ± 14.41	0.740	101.86 ± 12.49	0.239
		CT+CC	743	137.82 ± 20.10		85.38 ± 10.39		52.45 ± 15.09		102.86 ± 12.50	
**rs9651118**	**Additive**	CC	72	139.35 ± 20.29	0.649	86.90 ± 11.78	0.342	52.44 ± 14.67	0.808	102.86 ± 12.50	0.453
		TC	419	137.67 ± 20.44		84.98 ±10.21		52.69 ±15.56		102.53 ± 12.46	
		TT	551	137.11 ± 19.32		85.05 ± 10.66		52.06 ± 14.38		102.41 ± 12.44	
	**Dominant**	CC+TC	491	137.14 ± 19.66	0.515	85.26 ± 10.47	0.749	52.65 ± 15.42	0.522	102.80± 12.62	0.610
		TT	551	137.11 ± 19.32		85.05 ± 10.66		52.06 ± 14.38		102.41 ± 12.44	
	**Recessive**	CC	72	139.35 ± 20.29	0.410	86.90 ± 11.78	0.144	52.44 ± 14.67	0.951	102.86 ± 12.50	0.211
		TC+TT	970	137.35 ± 19.80		85.02 ± 10.46		52.33 ± 14.90		102.46 ± 12.44	
**rs117353193**	**Additive**	AA	1	130	0.620	80	0.889	50	0.474	97	0.817
		GA	96	135.71 ± 18.26		85.14 ± 10.39		50.57 ± 12.39		102.01 ± 12.33	
		GG	946	137.62 ± 19.98		85.12 ± 10.56		52.50 ± 15.10		102.62 ± 12.54	
	**Dominant**	AA+GA	97	135.65 ± 18.17	0.350	85.08 ± 10.35	0.973	50.57 ± 12.33	0.152	101.96 ± 12.18	0.620
		GG	946	137.62 ± 19.98		85.12 ± 10.56		52.50 ± 15.10		102.62 ± 12.54	
	**Recessive**	AA	1	130	-	80	-	50	-	97	-
		GA+GG	1042	137.45 ± 19.82		85.12 ± 10.54		52.32 ± 14.88		102.56 ± 12.50	
**rs9916351**	**Additive**	TT	123	140.91 ± 19.69	0.077	87.23 ± 11.58	**0.044^1^**	53.68 ± 13.59	0.432	105.11 ± 13.36	**0.035^1^**
		CT	487	137.69 ±20.40		85.08 ± 10.69		52.61 ± 15.37		102.62 ± 12.76	
		CC	434	136.35 ± 19.23		84.54 ± 10.01		51.81 ± 14.86		101.81 ± 11.90	
	**Dominant**	TT+CT	610	138.34 ± 20.28	0.110	85.51 ± 10.90	0.140	52.83 ± 15.02	0.279	103.12 ± 12.91	0.093
		CC	434	136.35 ± 19.23		84.54 ± 10.01		51.81 ± 14.86		101.81 ± 11.90	
	**Recessive**	TT	123	140.91 ± 19.69	**0.043^1^**	87.23 ± 11.58	**0.018^1^**	53.68 ± 13.59	0.313	105.11 ± 13.36	**0.017^1^**
		CT+CC	921	137.06 ± 19.86		84.83 ± 10.37		52.23 ± 15.13		102.24 ± 12.36	

Abbreviation: SD, standard deviation. ^1^Significant difference (*P*<0.05).

### Multiple linear regression analysis of five SNPs in genetic models

The results of multiple linear regression analysis of baseline covariates and genetic component on BP are shown in Table 7. In the recessive model, we found that *RNF213* rs9916351 had significant effects on SBP (*P*=0.025), DBP (*P*=0.017) and MAP (*P*=0.010) as a risk factor. However, there were no significant associations of rs1801131, rs1801133, rs9651118 and rs117353193 on BP.

**Table 7:** Multiple linear regression analysis of five SNPs in three genetic models

SNP	Genetic models	SBP		DBP		PP		MAP	
		β	*P*-value	β	*P-*value	β	*P*-value	β	*P*-value
**rs1801131**	**Additive**	1.238	0.347	0.079	0.913	1.159	0.275	0.468	0.570
	**Dominant**	1.763	0.241	0.344	0.678	1.420	0.241	0.807	0.392
	**Recessive**	−1.213	0.778	−1.955	0.407	0.742	0.830	−1.600	0.551
**rs1801133**	**Additive**	−0.627	0.503	−0.295	0.565	−0.332	0.660	−0.424	0.469
	**Dominant**	−1.954	0.230	−1.009	0.258	−0.945	0.471	−1.338	0.188
	**Recessive**	0.045	0.975	0.093	0.907	−0.048	0.968	0.045	0.961
**rs9651118**	**Additive**	0.806	0.457	0.492	0.412	0.314	0.719	0.617	0.365
	**Dominant**	0.854	0.524	0.714	0.335	0.139	0.897	0.785	0.351
	**Recessive**	1.522	0.571	0.151	0.919	1.370	0.527	0.634	0.707
**rs117353193**	**Additive**	2.698	0.266	0.227	0.865	2.471	0.207	1.026	0.500
	**Dominant**	2.666	0.285	0.084	0.951	2.582	0.199	0.924	0.554
	**Recessive**	9.356	0.604	8.155	0.412	1.202	0.934	8.279	0.464
**rs9916351**	**Additive**	1.137	0.258	0.851	0.124	0.287	0.724	0.939	0.135
	**Dominant**	0.049	0.972	0.370	0.624	0.321	0.772	0.258	0.764
	**Recessive**	4.547	**0.025^1^**	2.669	**0.017^1^**	1.878	0.253	3.279	**0.010^1^**

Abbreviation: β, partial regression coefficient. ^1^Significant difference (*P*<0.05).

## Discussion

Hypertension is a complex disease that comes about as a result of the interaction between genetic and environmental factors. Recently, numerous gene polymorphisms have been found to be associated with hypertension. The present study was designed to investigate the association of *MTHFR* (rs1801133, rs1801131, rs9651118), *TCN2* (rs117353193) and *RNF213* (rs9916351) gene variants with the susceptibility of hypertension and BP among the population of northeast in China.

In our study, we found that three *MTHFR* gene polymorphisms were not significantly associated with hypertension. Many genetic studies have shown that genetic variants in the *MTHFR* gene have been linked to CVDs, such as coronary heart disease [28,29], type 2 diabetes [30,31], ischemic stroke [32,33] and hypertension [34,35], but the clear mechanisms need to be further investigated. Markan et al. [36] reported that *MTHFR* rs1801133 and rs1801131 alleles and the co-occurrence of rs1801133 CT/rs1801131 CC genotypes were linked to increased risk of hypertension in the Indian population. Koupepidou et al. [37] also suggested that rs1801133 TT/CT and rs1801131 CC genotypes may be the risk factors for hypertensive renal sclerosis and chronic renal damage in hypertensive patients. Furthermore, a meta-analysis combining 5207 patients and 5383 control subjects indicated a significant association between the rs1801133 gene polymorphism and hypertension, which suggested that carriers of the T allele and TT genotype were more susceptible [38], but no significant association with rs1801131 was found. Additionally, more studies have reported that the *MTHFR* rs1801133 is an independent factor for hypertension in different ethnic groups [39–41]. A meta-analysis from 114 studies with 15411 cases and 21970 controls shown that the rs1801133 polymorphism was significantly associated with hypertension, and stratified analysis by ethnicity revealed a significant association among East Asians and Caucasians, but not among Latinos, Black Africans, and Indians and Sri Lankans. This shows the effect of ethnicity on the results, the differences in environmental exposures and genetic background among different populations might suggest potentially different pathways of BP regulation. However, for the rs1801131 polymorphism, no significant association was observed either in overall or subgroup analysis under all genetic models [24]. A number of studies have revealed that Hcy may be involved in the pathogenesis of hypertension, including that plasma Hcy could induce arteriolar constriction, renal dysfunction and increased sodium reabsorption [7]. Increased plasma Hcy levels contribute to vascular endothelium damage and promote oxidative stress, which lead to endothelial dysfunction and an imbalance of the antioxidant status [42–44]. Our study did not evaluate the level of Hcy. Inversely, Ravera et al. [45] found no association between rs1801133 and BP level among hypertensive patients. It was also reported that there is no significant association between rs1801133 and hypertension in Algerians [46]. In addition, no studies had reported the significant association between rs1801131 and hypertension. These results were consistent with ours. Meanwhile, we found that the statistical efficiency of these loci was above 80% in our power calculations, the results showed that: 98.9% power for rs1801131, 92.4% power for rs1801133, 97.6% power for rs9651118, 80.0% power for rs117353193, 100% power for rs9916351, this excluded the reason of insufficient sample size for the lack of association. We think the difference is more likely due to difference in the degree of ethnic heterogeneity. Regarding rs9651118, little studies on hypertension have been conducted up to date. Similarly vitamin B_12_ and folate are also involved in the metabolism of Hcy as nutritional factors. The former acts as a coenzyme in the catalytic synthesis of methionine with Hcy. *TCN2* is recognized as a specific plasma transporter to facilitate the cellular uptake of vitamin B_12_ by its receptor-mediated endocytosis [47]. Thus, *TCN2* gene polymorphisms have been considered as another genetic trait which may affect Hcy metabolism by modulating the bioavailability of vitamin B_12_ [48]. A genome-wide association study for MMD found that *TCN2* rs117353193 genotype frequency was significant difference between patients with normal Hcy levels and hyperhomocysteine [16], and this applies to *MTHFR* rs9651118 as well. In our study, we found that the risk of GG genotype carriers of rs117353193 is lower than that of GA+AA genotypes carriers after adjusting for confounding factors, which suggested that it may be a protective factor for hypertension. However, waistline and BMI also play important roles, so our results may be more influenced by environmental and genetic interactions. In addition, the mutant AA genotype carries are too few to compare with other genotypes carries. The relevant studies are so few and there is need for it to be further explored.

Regarding *RNF213*, it has been proved to be a susceptible gene to MMD [49], but it has also been reported to be involved in other vascular disorders such as coronary heart disease, hypertension, aneurysm and heterogeneous intracerebral vasculopathy [50–54]. One study showed a significant association of *RNF213* polymorphisms with SBP [53]. Our present study demonstrated that the homozygous TT genotype carriers of rs9916351 had significantly higher SBP, DBP and MAP levels than the CT or CC genotypes carriers, that is to say TT genotype might be a risk factor that is linked to increase the level of BP. Similarly, the results of multiple linear regression analysis also show that rs9916351 had significant effects on SBP, DBP and MAP as a risk factor, this further supports our conclusion. *MTHFR* rs9651118, *TCN2* rs117353193 and *RNF213* rs9916351 were recently revealed as the novel susceptibility loci for MMD by a genome-wide association study, no studies were conducted concerning the link to hypertension, and more evidences are needed to support our results.

Some limitations of our study should be mentioned. First, we did not get some of the relevant data in subjects, such as serum Hcy, vitamin B_12_ and folate levels. Second, the differences in results in different studies of different countries may also attribute to the ethnic differences. Furthermore, hypertension is a complex disease and is affected by both environmental and genetic factors. Some important characteristics were significantly different between the patients and the controls in our study, such as BMI, smoking, FBG and so on. Smoking is particularly known to exacerbate hypertension [55], we speculate that the higher proportion of female in the controls may have led to a higher incidence of smoking in the cases than in controls, which also had an impact on our results. Hypertension is a disease that seriously affects the health of all mankind, but at the same time, hypertension is also a controllable disease. While preventing its various risk factors, gene polymorphism also provides an important direction for us to study its pathogenesis and disease progress. However, our study mainly aimed to determine the association of gene polymorphisms on hypertension, the differences of basic data between the two groups had no effect on genotypes. Our study needs to be further studied on account of the consideration of above limitations.

## Conclusion

In summary, our study suggests that the *TCN2* rs117353193 gene polymorphism might serve as protective factor in hypertension, and the *RNF213* rs9916351 gene polymorphism might be an important risk factor that is linked to increase the level of BP among the population of northeast in China. Considering our relatively small sample size and narrow coverage, further studies are needed to confirm our results in the future.

## Abbreviations

BMI: body mass index
BP: blood pressure
CVD: cardiovascular disease
DBP: diastolic BP
FBG: fasting blood glucose
Hcy: homocysteine
LSD: least significant difference
MAP: mean arterial pressure
MMD: moyamoya disease
MTHFR: methylenetetrahydrofolate reductase gene
OR: odds ratio
PP: pulse pressure
RNF213: ring finger protein 213
RR: relative risk
SBP: systolic BP
SNK-q: student newman keuls
SNP: single nucleotide polymorphism
TC: total cholesterol
TCN2: transcobalamin*II*
TG: triglyceride
